# Are the effects of catch-and-release angling evident in changes to mRNA abundances related to metabolism, acid–base regulation and stress in lake trout (*Salvelinus namaycush)* gills?

**DOI:** 10.1093/conphys/coad065

**Published:** 2023-08-24

**Authors:** Simon W DePasquale, Bradley E Howell, Giulio Navarroli, Kenneth M Jeffries, Steven J Cooke, Sanoji Wijenayake, Jennifer D Jeffrey, Caleb T Hasler

**Affiliations:** Department of Biology, The University of Winnipeg, 515 Portage Avenue, Winnipeg, MB, Canada R3B 2E9; Department of Biology, The University of Winnipeg, 515 Portage Avenue, Winnipeg, MB, Canada R3B 2E9; Department of Biology, The University of Winnipeg, 515 Portage Avenue, Winnipeg, MB, Canada R3B 2E9; Department of Biological Sciences, University of Manitoba, 50 Sifton Road, Winnipeg, MB, Canada R3T 2N2; Department of Biology and Institute of Environmental and Interdisciplinary Science, Carleton University, 1125 Colonel By Drive, Ottawa, ON, Canada K1S 5B6; Department of Biology, The University of Winnipeg, 515 Portage Avenue, Winnipeg, MB, Canada R3B 2E9; Department of Biology, The University of Winnipeg, 515 Portage Avenue, Winnipeg, MB, Canada R3B 2E9; Department of Biology, The University of Winnipeg, 515 Portage Avenue, Winnipeg, MB, Canada R3B 2E9

**Keywords:** Physiology, recreational angling, recreational fisheries, salmonids, transcript level

## Abstract

Catch-and-release (C&R) angling is a conservation-oriented practice intended to reduce the impact recreational angling has on fish populations. Even though most recreationally angled fish are released, little is known about how C&R angling impacts fish at the cellular or tissue level. As the first to explore the impacts of C&R angling on mRNA abundances, our study aimed to identify how the stress of angling influenced metabolism, acid–base regulation and cellular stress in the gills of lake trout (*Salvelinus namaycush)*. Because gills are responsible for metabolic gas exchange, are crucial sites of acid–base homeostasis and respond to stressors quickly, we hypothesized that the relative mRNA abundance of genes related to these three physiological processes would be altered after angling. We took gill samples of live lake trout at 0, 2 or 48 h after fish were angled by rod and reel, and then used quantitative PCR (qPCR) to measure the relative abundance of nine candidate mRNA transcripts. *Heat shock protein 70* (*hsp70*) mRNA levels significantly increased over 5-fold 2 h after angling, indicating a potential activation of a cytoprotective response. However, contrary to our hypothesis, we observed no change in the relative mRNA abundance of genes related to metabolism or acid–base regulation in response to C&R angling within a 48-h period. As C&R angling can negatively impact fish populations, further use of transcript-level studies will allow us to understand the impact C&R has on specific tissues and improve our knowledge of how C&R influences overall fish health.

## Teaser

We examined the effects of catch-and-release (C&R) angling on mRNA abundances in lake trout gills. We observed some cellular stress response but no changes to metabolism or acid-base regulation. Further use of transcript-level approaches in this field will help elucidate how C&R angling negatively impacts fish health.

## Introduction

In some instances, recreational anglers can negatively influence wild fish populations by harvesting their catch (Post *et al.,* 2002; Lewin *et al.,* 2006). Thus, catch-and-release (C&R) angling has been promoted as a conservation-oriented practice to limit fishing mortality. Some anglers prefer to always practice C&R due to conservation ethic, but harvest regulations such as bag limits, slot sizes and no take fisheries require anglers to release non-harvestable/illegal fish. In Canada, for example, a 2015 survey estimated 2.5 million Canadians actively participated in fishing and that 66% of their catches were released (Fisheries and Oceans Canada, 2015). As over 100 million fish are caught and released each year in Canada (Fisheries and Oceans Canada, 2015), the efficacy of these laws dedicated to conserving fish populations rely on the assumption that fish are largely unimpacted by C&R (i.e. released fish survive to grow and reproduce; Wydoski, 1977).

Some fish die as a result of C&R, but there are also examples of recreational fisheries where mortality is relatively low, especially when anglers use good handling practices (Bartholomew and Bohnsack, 2005). However, even for fish that survive, there may be injuries, physiological alterations and behavioural impairments that collectively have the potential to impact fitness (reviewed in Arlinghaus *et al.,* 2007). Despite a large body of evidence showing the sublethal impacts of C&R, relatively few studies have explored C&R’s cellular- and tissue-specific effects and have only done so in a few cell/tissue types like red blood cells and white muscle (Kieffer *et al.,* 1995; Arlinghaus *et al.,* 2009). To date, no research has directly evaluated the impact of C&R on gill tissue, despite its importance to fish performance.

Changes in gene expression indicate how cellular processes of a specific tissue are regulated in response to a stressor. In the event of a stressor, select genes are differentially expressed allowing for a coordinated cellular response and return to homeostasis (e.g. Stears and Gullans, 2000). As an intermediate between DNA and protein, quantifying mRNA abundance serves as a proxy for changes in gene expression. Although a transcript-level approach has not yet been used to study the effects of C&R angling in fish, this approach has successfully been used to evaluate the effects of a wide variety of other stressors on fishes, including exercise to exhaustion (Wiseman *et al.,* 2007), hypoxia (Khansari *et al.,* 2018; Mu *et al.,* 2020; Ikert *et al.,* 2021; Liu *et al.,* 2022), hypercapnia (Edwards *et al.,* 2005; Georgalis *et al.,* 2006), heat exposure (Quinn *et al.,* 2011; Sun *et al.,* 2021) and a combined exhaustive exercise and air exposure stressor which simulated angling (Donaldson *et al.,* 2014). It is possible that C&R angling will produce similar cellular responses as the stressors listed above. For instance, fish strenuously exercise themselves during angling when they resist retrieval. After exhaustion, fish are then exposed to hypoxic or anoxic environments and often increased temperatures when they are handled out of the cooler water. Cumulatively, these stressors may enhance each other’s effects (Ferguson and Tufts, 1992; Beemelmanns *et al.,* 2021). Previous evidence of transcript-level effects in response to stressors similar in nature to those of C&R angling highlights this approach's potential as an effective tool for examining C&R responses.

Gills are the major site of metabolic gas exchange in teleost fishes (de Oliveira Ribeiro and Narciso, 2013) and are crucial sites of osmotic, ionic and acid–base homeostasis (Evans *et al.,* 2005). Transcript studies focused on stressors similar to C&R angling have found changes to many cellular processes in gills, including metabolism, acid–base regulation and the cellular stress response. More specifically, changes in the mRNA levels of genes involved in anaerobic glycolysis and processes of aerobic metabolism like the tricarboxylic acid (TCA) and the electron transport chain have been observed when fish are exposed to hypoxia (Mu *et al.,* 2020; Ikert *et al.,* 2021). Most metabolic mRNAs are upregulated because gills require additional energy to accelerate gas exchange under hypoxic conditions (Mu *et al.,* 2020). Additionally, mRNA levels of genes involved in acid–base regulation change during hypercapnia allowing the excretion of acid and retention of base equivalents (Edwards *et al.,* 2005; Georgalis *et al.,* 2006). Finally, transcript levels of heat shock proteins (HSPs) increase in response to a wide variety of cellular stress (Logan and Somero, 2011; Quinn *et al.,* 2011; Khansari *et al.,* 2018; Sun *et al.,* 2021). HSPs are molecular chaperones that retain the structure and function of a protein target. For instance, HSP70 protects nascent polypeptides and degraded proteins (Kiang and Tsokos, 1998), whereas HSP90 supports cytoskeleton and hormone receptor proteins (Csermely *et al.,* 1998). Collectively, HSPs maintain cellular homeostasis during exposure to stressful conditions (Sorenson *et al.,* 2003). As gills are in direct contact with the environment, they are often the first organ to respond to stress (Tiedke *et al.,* 2015), and therefore, gills are a preferred tissue for studying the acute effects of a stressor. Furthermore, gill sampling can be done non-lethally (Jeffries *et al.,* 2021), making gills a great option when studying wild fish.

As the first to use a transcript-level approach to study the effects of C&R angling, we aimed to identify how the stress of angling impacts three key cellular processes in the gills of lake trout (*Salvelinus namaycush*). We collected gill samples of wild-caught lake trout 0, 2 or 48 h after they were angled by rod and reel. We then used quantitative PCR (qPCR) to measure the relative mRNA abundance of nine candidate genes associated with metabolism, acid–base regulation and the cellular stress response. We hypothesized that transcript levels of genes associated with these three key cellular processes of gill tissue would be altered after C&R angling. Understanding the impacts of C&R angling on gill tissue will greatly improve our knowledge of how C&R angling impacts fish gills at the molecular level as well as overall fish health.

## Methods

### Field sampling

We handled and processed all fish according to protocols approved by the University of Winnipeg University Animal Care Committee (protocol #AE10491) and a Manitoban Provincial Scientific Collection Permit (#22758865). Fieldwork was completed on Treaty 5 Territory, within the homeland of the Swampy Cree of the Opaskwayak Cree Nation.

We angled lake trout (*Salvelinus namaycush*; derived from the Cree word nemekos, meaning ‘dweller of the deep’) from boat by rod and reel on Clearwater Lake, Manitoba (54.0251^°^N, 101.0327^°^W) between June 23 and July 18, 2022. We used 2.1 m medium-heavy spinning rods spooled with 9.1 kg line. We vertically jigged barbless lures/baits at or near the bottom of the lake (depth: 22.9 ± 7.6 m (mean ± SD)) where the mean water temperature and dissolved oxygen was 7.3 ± 1.2°C and 8.9 ± 1.8 mg/L, respectively (YSI 6050020 Pro 20 Dissolved Oxygen Meter, Xylem Inc., Yellow Springs, OH, USA). Specifically, we used a jigging spoon as an artificial lure or two baits. Both baits consisted of cut-up longnose sucker (*Catostomus catostomus*) presented either on a 10.2-cm tube jig or on a 2/0 treble hook. When we hooked-up to a fish, we recorded fight time (the time elapsed from hook-up to when we netted the fish). After landing the fish, we recorded the fork length (± 1 mm), total length (± 1 mm), weight (Berkley Digital Fish Scale—22.7 kg Pure Fishing Inc., Columbia, SC, USA), hooking location (external, inside the mouth or gullet), and level of bleeding at the hook site (none, slight or flow). We also recorded air exposure time as the time elapsed while we were sampling the fish. Before fish were released, we attached a coloured T-bar anchor tag to the left side of the dorsal fin to ensure we only sampled each fish once.

**Figure 1:**
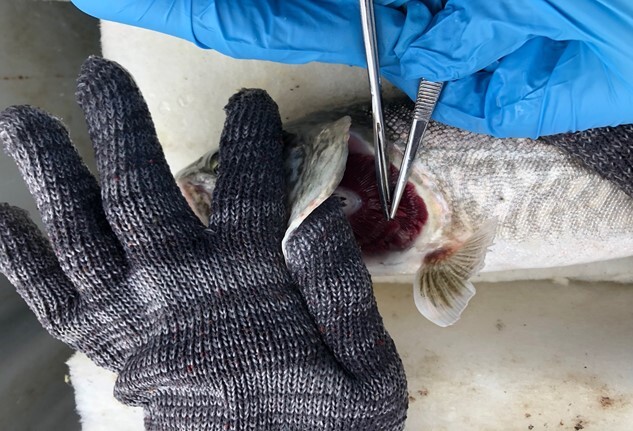
Non-lethal gill samples being excised from the distal tips of filaments on the second gill arch of lake trout (*Salvelinus namaycush*) after angling.

We collected gill samples non-lethally by excising an ~ 4-mm snippet of gill tissue from the distal tips of two or three filaments on the second gill arch (Jeffries *et al.,* 2021) (Figure 1). This technique is not invasive and does not affect fish survival or growth (McCormick, 1993). We immediately placed gill samples into RNA*later* solution (ThermoFisher Scientific, Waltham, MA, USA). We kept samples at 4°C for 24 h, and then froze them at −20°C until we could store them long term at −80°C. To determine if gill mRNA levels of various genes change after angling, we sampled each fish at one of three sampling periods, 0, 2 or 48 h after capture. Fish at 0 h (n = 8) (fork length: 503 ± 56 mm, total length: 551 ± 57 mm, weight: 1373 ± 442 g) were sampled immediately after capture, before the transcripts we were interested in have been shown to change in abundance (Wiseman *et al.,* 2007; Rimoldi *et al.,* 2009; Ikert *et al.,* 2021). We placed separate sets of fish in recovery bags (0.31 m diameter × 1.22 m length) (Fish Carrying Bags (Tubes); Dynamic Aqua Supply Ltd, Surrey, BC, CA) following capture. We suspended these bags 18.3 m below the surface of the water where the mean temperature and dissolved oxygen was 8.4 ± 1.4°C and 8.6 ± 1.0 mg/L, respectively (YSI 6050020 Pro 20 Dissolved Oxygen Meter). We retrieved fish from the recovery bags for gill sampling 2 h (n = 8) (fork length: 508 ± 27 mm, total length: 564 ± 23 mm, weight: 1438 ± 295 g) or 48 h (n = 8) (fork length: 534 ± 27 mm, total length: 583 ± 31 mm, weight: 1579 ± 293 g) after the initial capture. We also examined mortality for all fish that spent at least 48 h in a recovery bag (including fish that we did not run mRNA analysis on; n = 22).

### Laboratory analysis

We extracted total RNA from the gill samples using the RNeasy Plus Mini Kit (Qiagen, Toronto, ON, CA) following manufacturer's instructions. We determined the quantity, quality and integrity of RNA using a NanoDrop One (Thermo Fisher Scientific, Wilmington, DE, USA) and by visualization on a 1% w/v agarose gel. We converted 500 ng of total RNA into cDNA using High Capacity cDNA Reverse Transcription Kit (ThermoFisher Scientific) as per manufacturer's protocols. We determined the relative mRNA abundances of nine candidate genes using qPCR. We chose these targets based on their rapid induction in response to stressors similar to C&R angling (e.g. Liu *et al.,* 2022; Beemelmanns *et al.,* 2021; Sun *et al.,* 2021; Zarantoniello *et al.,* 2021; Esbaugh *et al.,* 2012; Georgalis *et al.,* 2006). We developed primer sets (Table 1) for each target and three reference genes using data available from GenBank (National Center for Biotechnology Information; NCBI) via Primer 3 Plus version 3.2.6 (Untergasser *et al.,* 2012), Primer Express 3.0.1 (Applied Biosystems, ThermoFisher Scientific) and Geneious Prime (Dotmatics, Boston, MA, USA). We used PowerUP SYBR Green Master Mix (Applied Biosystems, Thermo Fisher Scientific) and a QuantStudio 5 Real-Time qPCR System (Thermo Fisher Scientific) according to manufacturer's instructions. We generated standard curves for each primer set using serially diluted (1:4) cDNA pooled from all biological samples to optimize reaction compositions (efficiencies were between 88% and 107%; Table 1). We normalized target mRNA abundances to three reference genes and then expressed data in relation to the mean 0 h fish values using the 2^−ΔΔCT^ method (Livak and Schmittgen, 2001). Sampling period had no significant effect on mRNA levels for the reference genes.

**Table 1:** Oligonucleotide primers for quantitative PCR in lake trout (*Salvelinus namaycush*)

**Gene**	**Forward primer (5′—3′)**	**Reverse primer (5′—3′)**	**Product size (bp)**	**Efficiency (%)**
Metabolism				
*hk1*	TTGGCACTACTGCAGGTGAG	TCACATGTGCTGTCCAGACC	59	95.7
*cs*	TGTCCTTCAGCGCAGCTATG	TCCTGGTTAGCCAGTCCATGA	61	92.1
*cco5b*	GTGTGGTTCTGGCTCCATCA	TTTAGTGGGGCAGCTCATGG	91	92.3
Acid–base regulation				
*nka-a1*	TGACGCTGACACCACAGAGAA	CCAGGTGGCAGAGCTTTTGT	61	93.3
*cahz*	CACAGCTGGGGATATGGACC	CTGCCTGGGTCCATTAGCAA	78	107.1
*nhe1*	GCCAAGCGCTCCATCAAT	CAATGCCAGTCAGCAGATGGT	64	97.5
Cellular stress response				
*hsp70*	GAACACTGTCCTCCAGCTCC	CCCTGAAGAGGTCGGAACAC	120	88.2
*hsp90aa1*	CAACTCCACCATGGGCTACA	GGGTGGGTTGGGTTGATCTC	60	99.5
*hsp11b*	GCAACACACTTGCGCTTCA	CCCTGTGTACCGAGACAAAATG	61	97.7
Reference targets				
*rps9*	TCTCCCTGCGTTCACCATAC	GCCCTTCTTGGCATTCTTTC	68	101.4
*rps6*	CCAAAAGGCGCCGTCTCT	GGCTGGACTCAGATTTGGATGT	57	106.0
*rpl13a*	CACTGGAGAGGCTGAAGGTG	GTGGGCTTCAGACGGACAAT	103	106.3

hk1, hexokinase 1; cs, citrate synthase; cco5b, cytochrome c oxidase 5b; nka-a1, sodium potassium transporting ATPase alpha 1; cahz, carbonic anhydrase; nhe1; sodium-hydrogen exchanger 1; hsp70, heat shock protein 70; hsp90aa1, heat shock protein 90 alpha 1; hsp11b, heat shock protein 11b; rps9, 40S ribosomal protein S9; rps6, 40S ribosomal protein S6; rpl13a, ribosomal protein L13a.

### Statistical analysis

We determined differences in the relative mRNA abundances of target genes among sampling periods using a one-way analysis of variance (ANOVA), followed by a Tukey's honestly significant difference (HSD) test. The assumptions of equal variance and normality were tested for each target using Levene's and Shapiro–Wilk's tests, respectively. We performed all statistical tests using R version 4.0.2 (R Core Team, 2019) with significance defined as α < 0.05.

## Results

**Figure 2:**
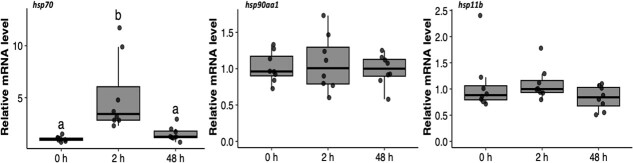
Relative mRNA levels of genes associated with cellular stress (heat shock proteins) in the gills of lake trout (*Salvelinus namaycush*) sampled 0, 2 or 48 h after being angled. Letters represent significant differences between gill sampling times (Tukey’s HSD post hoc test). Horizontal bars in the boxplot represent the median response value and the 75% and 25% quartiles. Whiskers represent ±1.5 times the interquartile range, and each dot represents an individual response value (n = 8). *hsp70, heat shock protein 70; hsp90aa1, heat shock protein 90 alpha 1; hsp11b, heat shock protein 11b.*

**Figure 3:**
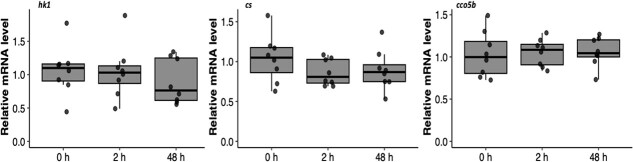
Relative mRNA levels of genes associated with metabolism in the gills of lake trout (*Salvelinus namaycush*) sampled 0, 2 or 48 h after being angled. Horizontal bars in the boxplot represent the median response value and the 75% and 25% quartiles. Whiskers represent ±1.5 times the interquartile range, and each dot represents an individual response value (n = 8). *hk1, hexokinase 1; cs, citrate synthase; cco5b, cytochrome c oxidase 5b.*

**Figure 4:**
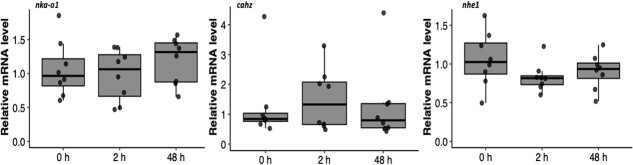
Relative mRNA levels of genes associated with acid–base regulation in the gills of lake trout (*Salvelinus namaycush*) sampled 0, 2 or 48 h after being angled. Horizontal bars in the boxplot represent the median response value and the 75% and 25% quartiles. Whiskers represent ±1.5 times the interquartile range, and each dot represents an individual response value (n = 8). *nka-a1, sodium potassium transporting ATPase alpha 1; cahz, carbonic anhydrase; nhe1, sodium-hydrogen exchanger 1.*

We held some fish in recovery bags for longer than 48 h because weather prevented us from boating out to our bags. We did not gill sample these fish, but report that of the 22 fish that we held in recovery bags for ≥48 h, we observed four mortalities (mortality rate = 18%). We analysed mRNA levels in gill samples taken from 24 fish. The mean fight time was 32 ± 12 s (range: 13 to 60 s) and the mean air exposure time was 125 ± 57 s (range: 45 to 258 s). Angling significantly altered *heat shock protein 70* mRNA relative transcript abundance (*hsp70;* one-way ANOVA: *F*_2,21_ = 23.28, *P* < 0.001). We observed that *hsp70* levels significantly increased by approximately 5-fold 2 h after angling and then returned to near 0 h levels 48 h after angling (Figure 2). We observed no change in mRNA levels of *heat shock protein 90 alpha 1* (*hsp90aa1*; *F*_2,21_ = 0.10, *P* = 0.91) or *heat shock protein 11b* (*hsp11b; F*_2,21_ = 1.86, *P* = 0.18) after angling (Figure 2). There were minimal transcriptional changes in *hexokinase 1* (*hk1*; *F*_2,21_ = 0.48, *P* = 0.62), *citrate synthase* (*cs; F*_2,21_ = 0.98, *P* = 0.39) and *cytochrome c oxidase 5b* (*cco5b; F*_2,21_ = 0.11, *P* = 0.89) after angling (Figure 3). Similarly, the relative mRNA abundances of *sodium potassium transporting ATPase alpha 1* (*nka-a1*; *F*_2,21_ = 0.74, *P* = 0.49), *carbonic anhydrase* (*cahz; F*_2,21_ = 0.23, *P* = 0.79) and *sodium-hydrogen exchanger 1* (*nhe1; F*_2,21_ = 1.02, *P* = 0.38) did not change in response to angling (Figure 4).

## Discussion

We examined the consequences of C&R angling on molecular markers in the gills of lake trout. Overall, contrary to our hypothesis, there was limited evidence for a meaningful impact of C&R angling on the molecular factors we quantified. We observed no change in the relative transcript abundances of metabolic or acid–base regulatory mRNAs, suggesting that C&R angling has little impact on these cellular processes in lake trout gills, when fight times are less than 60 s and air exposure times less than 258 s. While we observed no change in *hsp90aa1* and *hsp11b*, *hsp70* mRNA levels significantly increased by over 5-fold 2 h after C&R angling, indicating a potential activation of cytoprotective responses in lake trout gills.

### Cellular stress

Overall, our results from the *hsp* mRNAs showed some activation of a cellular stress response. Heat shock proteins are ubiquitously expressed molecular chaperones, playing roles in protein transport, folding, assembly and degradation (Sorenson *et al.,* 2003). In response to a stressor, cells upregulate HSPs to protect protein structure and function. A multitude of stressors are known to increase HSP levels. Of relevance to the responses to C&R angling, previous work on fish have found that *hsp* mRNAs levels increase in response to heat exposure (Beemelmanns *et al.,* 2021; Sun *et al.,* 2021; Zarantoniello *et al.,* 2021; Quinn *et al.,* 2011; Logan and Somero, 2011) and exposure to hypoxic conditions (Khansari *et al.,* 2018; Beemelmanns *et al.,* 2021). Consistent with these studies, we found that *hsp70* relative transcript abundance was significantly increased 2 h after angling, suggesting HSP70 expression may have been induced to help conserve protein structure and function after exposure to C&R angling (Sorenson *et al.,* 2003). Interestingly, we observed that *hsp70* mRNA levels recovered at some point between 2 and 48 h after angling. Incidences of mortality following C&R angling have been shown to be greatest within the first 48 h (Mongillo, 1984; Hall *et al.,* 2009), and recovery of *hsp70* transcript levels here also supports that some of the impacts of C&R angling might subside within this timeframe.

The recovery of *hsp70* mRNA and our observation of similar mRNA levels between control and 48 h fish for the other markers also suggest that the recovery bags we used did not impact fish metabolism, acid–base regulation or cellular stress. Recovery bags are a simple and inexpensive tool that have been used with some success to enable the recovery of wild Pacific salmon (*Oncorhynchus keta*) (Donaldson *et al.,* 2013) and bonefish (*Albula* spp.) (Brownscombe *et al.,* 2013) following various fisheries stressors. Using these recovery bags, we observed a mortality rate of 18% 48 h post-angling. This mortality rate is similar to those previously reported in the literature for lake trout (Persons and Hirsch, 1994; Dextrase and Ball, 1991), also supporting these recovery bags as a good option to hold fish that are exhausted (for short periods) while they recover.

Differences in the molecular chaperone activities of the HSPs may help explain why only *hsp70* mRNA levels responded to C&R angling. As a chaperone, HSP70 targets include nascent polypeptide chains as well as degraded or altered proteins (Kiang and Tsokos, 1998). Conversely, HSP90 supports cytoskeleton and hormone receptor proteins and in most cases, it alone cannot help refold partially denatured proteins (Csermely *et al.,* 1998). Instead, HSP90 requires other chaperones like HSP70 to aid in successfully refolding a protein (Freeman and Morimoto, 1996). In comparison to HSP90, the independent role HSP70 plays in refolding proteins may indicate why it was a more sensitive indicator of C&R angling induced stress. However, studies examining the effect of stressors on multiple HSPs typically find unity in the way they respond (Quinn *et al.,* 2011; Beemelmanns *et al.,* 2021; Sun *et al.,* 2021). Because transcript levels of *hsp90aa1* and *hsp11b* were not observed to change, this could mean that the C&R angling stressor used in our study did not induce large changes in the cellular stress response (Donaldson *et al.,* 2014) and that these C&R practices are appropriate for lake trout in the contexts studied here.

### Metabolism

Contrary to our hypothesis, we observed no changes in the mRNA levels of genes associated with metabolism examined in the present study. Acute tissue hypoxia, induced by air exposure during C&R, should increase both aerobic and anaerobic metabolism in gill tissue (Mu *et al.,* 2020). Aerobic metabolism increases as an initial response to acute hypoxia, to maximize the flux of oxygen from the environment to the gills (Ngan and Wang, 2009). When the rate of oxidative phosphorylation is limited by low oxygen availability, the rate of anaerobic glycolysis also increases to meet energy demands. Likely, acute hypoxia via a 45- to 258-s air exposure may have been too brief to elicit upregulations in transcript levels of *hk1*, *cco5b* and *cs,* which are involved in glycolysis, the electron transport chain and the TCA cycle, respectively. A brief, 180 s, air exposure also yielded mostly insignificant impacts on the mRNA abundances of metabolic genes in the livers of rainbow trout (*Oncorhynchus mykiss*) and brook trout (*Salvelinus fontinalis*) (Ikert *et al.,* 2021). Conversely, studies that have found significant changes in glycolytic genes and TCA cycle associated genes in the gills of largemouth bass (*Micropterus salmoides*) (Liu *et al.,* 2022) and yellow croaker (*Larimichthys crocea*) (Mu *et al.,* 2020) exposed to hypoxia for at least 10 min. Exhaustive exercise should also impact metabolism, where increases in anaerobic metabolism have been observed in strenuously exercised rainbow trout (Wiseman *et al.,* 2007; Lopez-Patino *et al.,* 2014). Thus, it is likely that changes to inducible metabolic mRNA markers would only occur during C&R if an angler held a lake trout out of water for longer than the 45- to 258-s air exposure that we used, and/or fought lake trout for longer than our maximum 60 s fight time.

### Acid–base regulation

We also observed no change in the mRNA levels of genes associated with acid–base regulation, which might indicate that C&R angling did not induce severe or prolonged hypercapnia (respiratory acidosis). Whereas it is well known that C&R induces metabolic acidosis in salmonids and other fish because of increased anaerobic production of lactate (e.g. Arlinghaus *et al.,* 2009; Donaldson *et al.,* 2014; Twardek *et al.,* 2018; Card and Hasler, 2021), less known is the extent to which C&R causes hypercapnia. The combination of strenuous exercise and air exposure during C&R should induce some level of hypercapnia (Ferguson and Tufts, 1992). However, to date, few studies have documented this relationship and have only done so in black basses (*Micropterus spp.*, Kieffer *et al.,* 1995; Dinken *et al.,* 2022). Furthermore, Gallagher *et al.* (2014) found that fight time could not predict blood pCO_2_ levels in several shark species. Our study offers additional evidence that some fish species may not experience severe hypercapnia during C&R, as we found no changes in *cahz*, *nhe1* and *nka-a1* mRNA levels at any sampling point. During hypercapnia, gill cytosolic *cahz* mRNA is significantly upregulated within 3 h (Georgalis *et al.,* 2006), because it converts CO_2_ into acid–base equivalents for excretion (Sobotka and Kann, 1941). Additionally, reductions in *nhe1* mRNA levels (Rimoldi *et al.,* 2009) and NKA activity (Esbaugh *et al.,* 2012) have been shown in response to acute (1 and 24 h, respectively) hypercapnia. Localized to the basolateral membrane, NHE1 pumps protons into the gill serosa, thus a reduction in NHE1 activity combined with increased proton excretion into the external environment maximizes net acid excretion (Claiborne *et al.,* 1999). NKA is also basolaterally located, and its reduced activity increases cytoplasmic Na^+^ favouring the uptake of HCO_3_^−^ (Esbaugh *et al.,* 2012). Additionally, hypercapnia may have occurred, but was not prolonged or severe enough to induce changes in mRNA abundances. For instance, Georgalis *et al.* (2006) did not observe changes in gill cytosolic *cahz* until fish were exposed to continuous hypercapnia for 3 h. Considering that fight times in our experiment were less than 60 s and air exposure times less than 258 s, recovery to hypercapnia may have been quick or hypercapnia too weak and short-lived to change the levels of acid–base regulatory mRNAs.

### Future direction of transcript-level C&R studies

Analysis of fish blood, the most common physiological tool used to assess impacts of C&R angling (Cooke *et al.,* 2013), has shown altered metabolism and a cellular stress response following angling. Changes in blood metrics such as lactate and pH indicate that fish experienced increased anaerobic metabolism (e.g. Gustaveson *et al.,* 1991; Twardek *et al.,* 2018; Card and Hasler, 2021). A metabolic acidosis, shown by reductions in blood pH occurs largely because of increased anaerobic production of lactate (Wood, 1991). Using a transcript-level approach, we did not find that C&R angling altered metabolic or the acid–base regulatory mRNA levels after angling. However, it is possible that basal levels of these proteins were sufficient to respond to the stress of C&R angling without necessitating transcript-level changes. It is also possible that lake trout in our study experienced increased anaerobic metabolism, undetected by the limited mRNA markers used in the study. For instance, full transcriptome studies on fish have found that only a small percentage (<10%) of metabolic mRNA markers change in response to hypoxia (Gracey *et al.,* 2001; Everett *et al.,* 2012). Elevated blood cortisol has shown that fish experience a cellular stress response after angling (Hall *et al.,* 2009). Similarly, in our study, increases in mRNA levels of *hsp70* in response to C&R angling reflected activation of the cellular stress response.

Future C&R angling transcript-level studies should examine mRNA levels in red blood cells, use a larger sample size and sample at more timepoints. Red blood cells, like gill tissue, can be obtained non-lethally (Jeffries *et al.,* 2021). Because both cell types work together to move respiratory gases and expel wastes, transcript-level responses of gill and red blood cells to a stressor are often similar (Akbarzadeh *et al.,* 2018). Furthermore, blood contamination within the gill tissue sample could also lead to similar transcript-level responses between the cell types. However, because it is unknown how gill tissue responds to angling, and ample work has deduced how blood metrics change, future studies exploring how angling impacts mRNA abundances in blood will help confirm our observations of minimal transcript-level changes to fish metabolism, acid–base regulation and stress. Controlling the conditions, a wild fish experiences prior to C&R angling is impossible. These varying conditions could lead to mRNA transcript-level discrepancies between fish, masking subtle patterns of change. In the future, use of a larger sample size (>20 fish) might help to elucidate subtle responses. We only gill sampled fish one of three different time points. Although transcript levels of certain mRNAs often change in the first 1 to 3 h (Quinn *et al.,* 2011; Rimoldi *et al.,* 2009; Georgalis *et al.,* 2006), we potentially would have missed any transcript level changes that occurred after 2 h, but recovered by 48 h. We recommend that future studies sample at least one more time point between 2 and 48 h.

Currently, the biggest challenge associated with using a transcript-level approach in C&R science is a lack of directly comparable literature. Although many studies use stressors similar in nature to C&R, they subject fish to stressors of much higher duration or strength than angling does. Thus, currently, we can only conclude that C&R has less of an impact on metabolic or acid–base regulatory mRNA levels than does a 10-min hypoxia (Mu *et al.,* 2020) or a 3-h hypercapnia exposure (Georgalis *et al.,* 2006). However, once this technique is used more within C&R science and other mRNA targets that change in response to C&R are identified, more meaningful comparisons could be made. For instance, we identified some initiation of a cellular stress response, via changes to *hsp70* mRNA levels. Thus, using *hsp70* transcript levels, other studies could now compare the impacts C&R angling has on cellular stress between species of fish or different angling techniques. Conversely, we found no impact on metabolic or acid–base regulatory mRNA levels. Future research might be able to identify which mRNA markers change in response to these processes or find that they do change when studying a different fish species or when using different angling techniques. Further incorporation of transcript-level approaches that provide insight into how specific fish tissues are affected by angling will yield mechanistic insight needed to develop best practices for C&R.
